# Comparison of Macular Integrity Assessment (MAIA ™), MP-3, and the Humphrey Field Analyzer in the Evaluation of the Relationship between the Structure and Function of the Macula

**DOI:** 10.1371/journal.pone.0151000

**Published:** 2016-03-14

**Authors:** Kazuyuki Hirooka, Kana Misaki, Eri Nitta, Kaori Ukegawa, Shino Sato, Akitaka Tsujikawa

**Affiliations:** Department of Ophthalmology, Kagawa University Faculty of Medicine, 1750–1 Ikenobe, Miki, Kagawa 761–0793, Japan; The University of Melbourne, AUSTRALIA

## Abstract

**Purpose:**

This study was conducted in order to compare relationships between the macular visual field (VF) mean sensitivity measured by MAIA^TM^ (Macular Integrity Assessment), MP-3, or Humphry field analyzer (HFA) and the ganglion cell and inner plexiform layer (GCA) thicknesses.

**Methods:**

This cross-sectional study examined 73 glaucoma patients and 19 normal subjects. All subjects underwent measurements for GCA thickness by Cirrus HD-OCT and static threshold perimetry using MAIA^TM^, MP-3, or HFA. VF and OCT in the retinal view were used to examine both the global relationship between the VF sensitivity and GCA thickness, and the superior hemiretina and inferior hemiretina. The relationship between the GCA thickness and macular sensitivity was examined by Spearman correlation analysis.

**Results:**

For each instrument, statistically significant macular VF sensitivity (dB) and GCA thickness relationships were observed using the decibel scale (R = 0.547–0.687, all *P* < 0.001). The highest correlation for the global (R = 0.682) and the superior hemiretina (R = 0.594) GCA thickness-VF mean sensitivity was observed by the HFA. The highest correlation for the inferior hemiretina (R = 0.687) GCA thickness-VF mean sensitivity was observed by the MP-3. Among the three VF measurement instruments, however, no significant differences were found for the structure-function relationships.

**Conclusions:**

All three VF measurement instruments found similar structure-function relationships in the central VF.

## Introduction

Although macular visual field (VF) defects have been thought to occur in later stages, recent studies have shown to occur during the early stages of glaucoma [1–3]. As central VF defects can adversely affect driving performance and reading ability, clinicians need to carefully evaluate VF defects that occur near the fixation point in glaucoma patients [4,5]. With the implementation of new segmentation algorithms, optical coherence tomography (OCT) can now be used to visualize and measure the individual retinal layers in the macular region [6–8]. Ganglion cell analysis (GCA) can be performed using OCT, which measures the retinal ganglion cell (RGC) or the RGC + inner plexiform layer (IPL) thicknesses [7,9,10]. Using the Cirrus HD-OCT GCA algorithm (Carl Zeiss Meditec, Dublin, CA), Mwanza et al. [11,12] were able to detect and measure the inner macular layers, which contained the ganglion cell layer and the IPL. When using this technique, they additionally found there was excellent intervisit reproducibility [11,12].

Since 2009, microperimetry field advancements (Macular Integrity Assessment: MAIA ^TM^; CenterVue, Padova, Italy) have facilitated the assessment of the macular function through accurate measurements of the visual field sensitivity, fixation stability and the site of fixation [13]. The MP-3 (Nidek Technologies Srl, Padova, Italy) is a newly developed instrument that makes it possible to perform fundus perimetry. An advantage of using MAIA^TM^ or the MP-3 is that these devices can provide a broader tracking system with a clear and detailed retinal image. Due to the quality of these images, this results in a higher reliability. Furthermore, MAIA^TM^ and the MP-3 both provide precise eye tracking systems that can be used to perform a quantitative analysis of a patient’s fixation in terms of stability and location [13,14].

The Humphrey Field Analyzer (HFA; Carl Zeiss Meditec, Dublin, CA) has been shown to have high test-retest variability. Because of this, HFA cannot reliably identify VF progression, in addition to only being able to document a poor relationship between the structure and function [15]. One of the major factors in this test-retest variability involves fixation errors [16]. Microperimetry utilizes precise eye tracking systems that are able to improve eye fixation stabilization. Thus, the use of this technique can lead to improved calculations of the correlation coefficient, thereby making it possible to better define the relationship between the structure and function.

Spectral-domain OCT has been used in multiple studies to examine the macula and the relationship between structural and functional damage [9,10]. This method improves the detection of the presence and progression of glaucomatous damage [15]. We previously used Cirrus HD-OCT to measure the GCA thickness and demonstrated that there were statistically significant structure-function associations with the MAIA^TM^-measured central VF [10]. The HFA has been used as the standard tool for performing a functional assessment of the visual performance. In addition, the HFA is the clinical standard device for evaluating glaucoma. In our current investigation, we attempted to further evaluate which perimetry best reflects the structure-function relationship in the central VF, as we believe the results of such a study would be of great interest to both clinicians and researchers in this field.

The overall aim of this study was to compare the measurements of the macular VF mean sensitivity that were made by the MAIA^TM^, MP-3, and HFA instruments, and then determine if there was any potential relationships between these measurements and the GCA thicknesses.

## Materials and Methods

This study was performed at Kagawa University Hospital, with all of the patients examined from November 2014 through March 2015. The macular inner structure thickness was examined at each patient visit through the use of the GCA algorithm. Patients were also examined by MAIA^TM^, MP-3 and HFA, with the results used for calculating the static threshold perimetry. Prior to the examinations, all of the subjects were given a detailed explanation of the study, and then asked to sign an informed consent form in accordance with the principles embodied in the Declaration of Helsinki. The Institutional Review Board of the Kagawa University Faculty of Medicine approved the study protocol. The normal controls used in this study were volunteers who were recruited from subjects visiting our outpatient clinics, were spouses or friends of the enrolled patients, or were members of our hospital staff.

A complete ophthalmic examination was performed at the start of the study. The tests performed in all subjects included visual acuity testing with refraction, intraocular pressure (IOP), gonioscopy examinations, and dilated fundus examination with stereoscopic biomicroscopy of the optic nerve head using slit lamp and indirect ophthalmoscopy. Subjects with a best-corrected visual acuity of 20/40 or better, a spherical error within a range between +4.0 and -6.0 diopters, a cylinder within ± 2.0 diopters, and open angles (grade 3 and 4 according to the Shaffer grading system) were included in the study. However, any subjects with a history of retinal pathology or neurologic disease, retinal laser procedure, or either retinal or intraocular surgery, were excluded from the study. After being enrolled, one eye in each subject was randomly chosen for inclusion in the study. Control subjects were required to have an IOP ≤ 21 mmHg, no history of retinal pathology, and a normal visual field. In order to be defined as glaucoma, eyes had to exhibit the following structural glaucomatous changes. First, the vertical cup-disc asymmetry between the fellow eyes needed to be ≥ 0.2. Second, the cup-to-disc ratio had to be ≥ 0.6, and the eye needed to exhibit neuroretinal rim narrowing, notches, localized pallor, or RNFL defects with glaucomatous VF loss in the corresponding hemifield. To be defined as glaucomatous VF, the subject had to have a glaucoma hemifield test (GHT) outside of the normal limits on at least two consecutive baseline tests, with at least three contiguous test points within the same hemifield on the pattern deviation plot at *P* < 1%, and at least one at *P* < 0.5%, excluding those points that were on the edge of the field or that were directly above and below the blind spot.

### Cirrus HD-OCT Imaging

The imaging protocol for the Cirrus HD-OCT device (version 6.0 of the GCA algorithm) has been previously described [10]. Briefly, this protocol uses a macular cube with 200 (vertical) × 200 (horizontal) scans that are performed 1024 times within a cube that measures 6 × 6 × 2 mm [10]. The macular GCA thickness was automatically measured within a 14.13 mm^2^ elliptical annulus area centered on the fovea through the use of the GCA analysis algorithm. For all of the data analyses, only good quality scans were used. A good quality scan was defined as a scan that had a signal strength ≥ 6, was without retinal nerve fiber layer (RNFL) discontinuity or misalignment, had no involuntary saccade or blinking artifacts, and during a careful visual inspection, the image showed an absence of algorithm segmentation failure. In order to create a region of interest that lies within the intraretinal layers, the input image data are initially segmented using the existing Cirrus inner limiting membrane (ILM) and retinal pigment epithelium (RPE) segmentation algorithms [11,12]. These algorithms are able to identify the RNFL layer, the GCA layer, and the outer retinal layer. The GCA layer is the region that is found between the outer boundary of the RNFL and the outer boundary of the IPL. This area includes both the RGC layer and the IPL. By using a graph-based algorithm to identify each layer, the segmentation procedure is able to operate in three dimensions. Description of how the algorithm specifically operates has been previously presented in detail [11,12]. For the nine thicknesses calculated by the algorithm, all measurements were performed within the annular area that is centered on the fovea. The measurements taken included the global, superior hemifield and inferior hemifield averages, along with the six sectoral values of the respective layers (superotemporal, superior, superonasal, inferotemporal, inferior, and inferonasal).

### Microperimetry Examinations

MAIA^TM^ measurements were performed without any dilation of the pupil in a dim room. The 4–2 threshold strategy was used during the MAIA^TM^ examinations. The specific parameters used included a 68-stimuli grid covering the central 10° of the retina, with a fixation target composed of a red circle with a 1° diameter. A standard Goldmann III stimulus size was used, with the background luminance set at 4 asb, while the maximum luminance was set at 1,000 asb. The stimulus dynamic range for the device was 36 dB. One of the benefits of the MAIA^TM^ device is that it makes it possible to determine which of the fixation stabilities are stable. As result, we were able to perform our data analysis using only stable fixation tests.

Similar to the MAIA^TM^ measurements, we also performed the MP-3 examinations in a dim room. However, since this instrument requires at least a 4 mm pupil diameter, some of the patients had to undergo pupil dilation prior to this examination. The parameters for the MP-3 device were similar to those used for the MAIA^TM^ device. Thus, we used the 4–2 threshold strategy, with a 68-stimuli grid covering the central 10° of the retina, and a fixation target that was composed of a red circle with a 1° diameter. This examination also used a standard Goldmann III stimulus size, with the background and maximum luminance set at 31.4 and 10,000 asb, respectively. The stimulus dynamic range for the MP-3 was 34 dB. Similar to that for the MAIA^TM^ device, the MP-3 device was also able to determine which of the fixation stabilities were stable. Thus, our analyses only used the stable fixation tests.

### Visual Sensitivity of the 10–2 HFA

We used static automated white-on-white threshold perimetry (HFA; 10–2 Swedish Interactive Threshold Algorithm Standard test) to examine the visual sensitivity. When the fixation losses and the false-positive/false-negative rates were less than 20%, the VF results were considered to be reliable. Our subsequent analyses only use the reliable test data.

### Mapping Structure to Function

**Fig 1:**
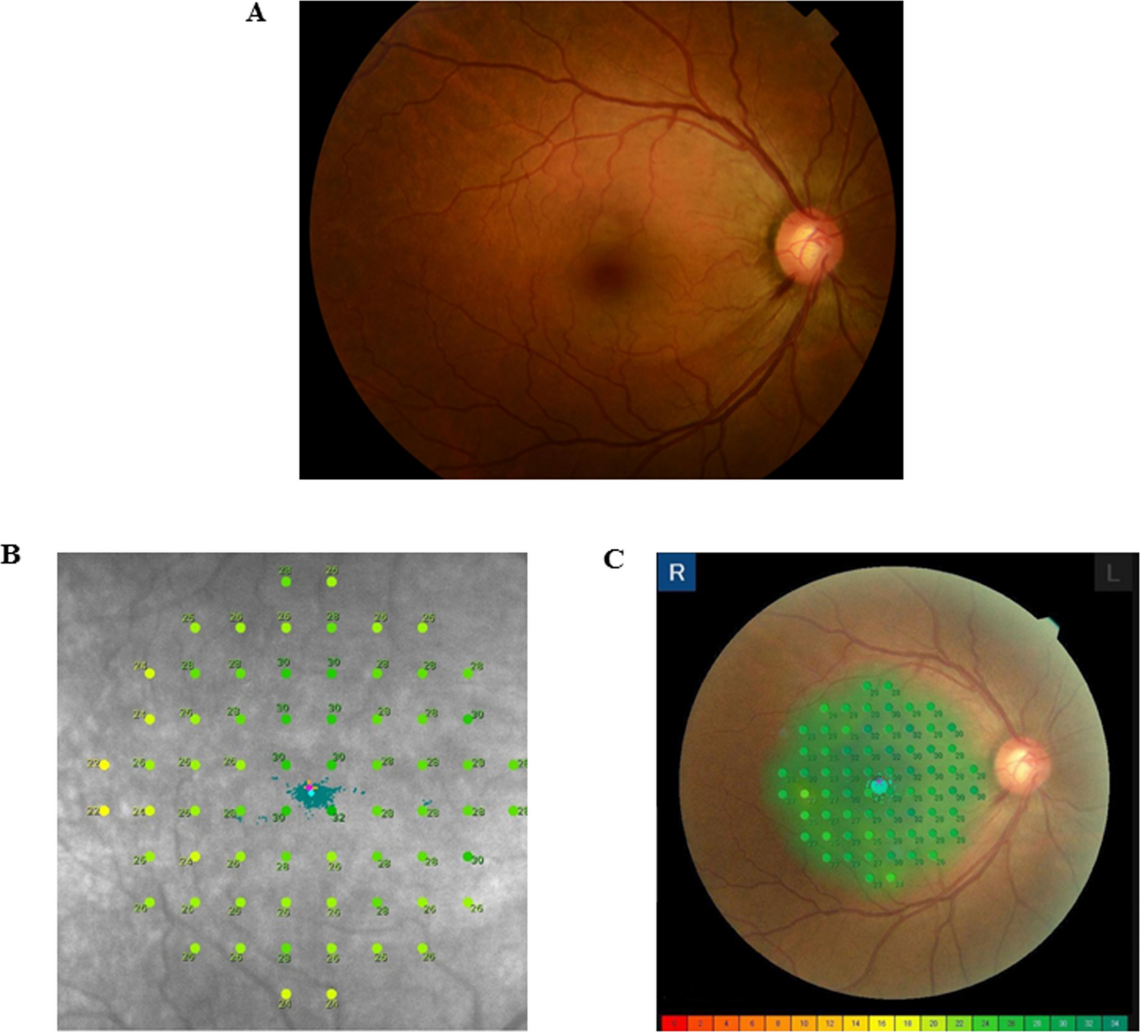
Detection of the macular sensitivity in the right eye fundus image of a 47-year-old patient with primary open-angle glaucoma (POAG). Right eye fundus photo (A). Microperimetry results for the differential light thresholds that ranged from 0 to 36 dB with MAIA^TM^ (B) and from 0 to 34 dB with MP-3 (C) are shown.

**Fig 2:**
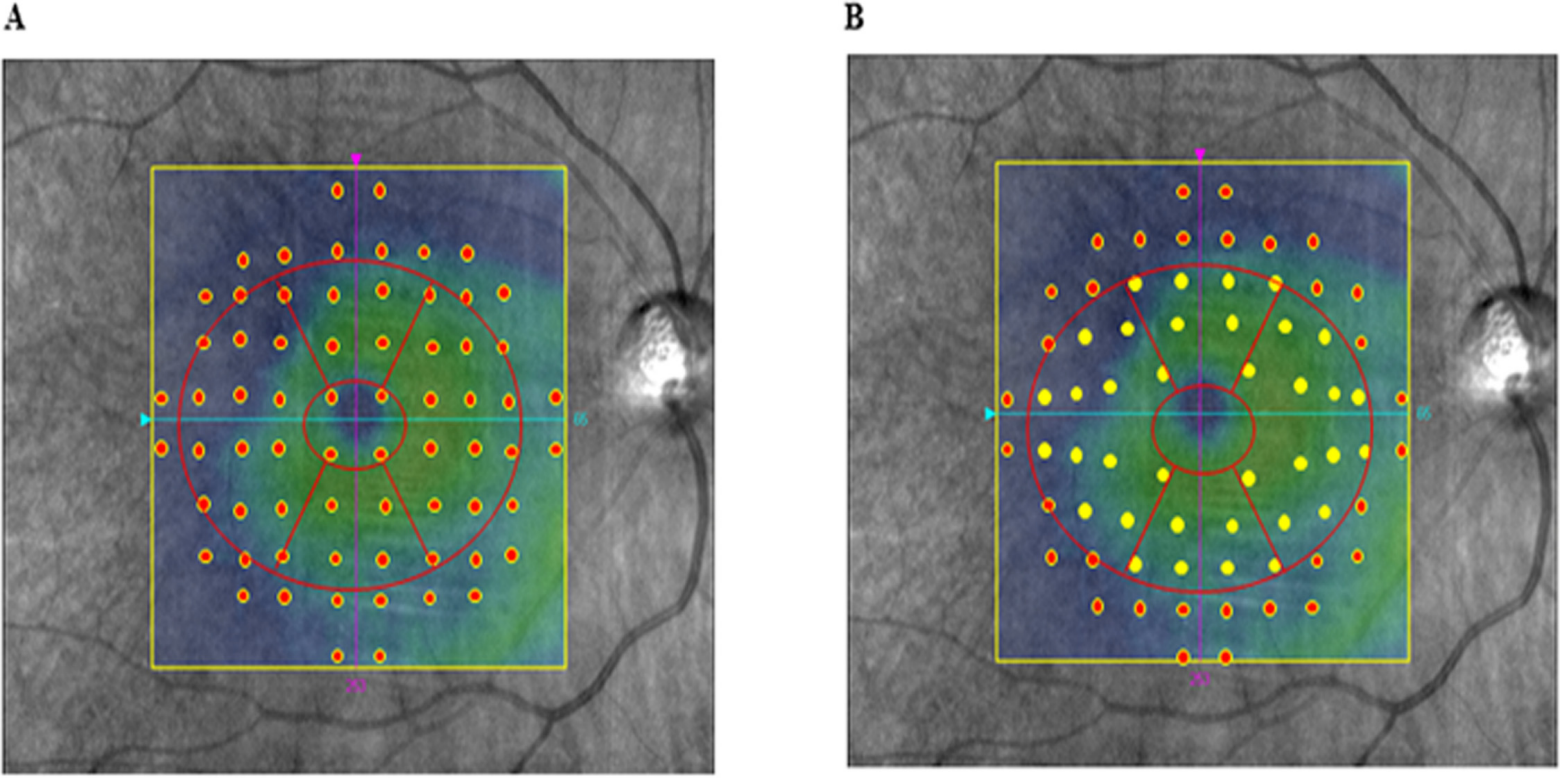
Image showing the ganglion cell analyzer and before and after the adjustment for the retinal ganglion cell displacement. (A) The 10–2 VF displaced points. (B) The 10–2 VF displaced points corresponded to the RGC locations. These locations were based on a model that was derived from a previous histological analysis.^13^ After dividing the GCA thickness areas into the upper and lower hemifield sectors, either MAIA^TM^, MP-3, or HFA was used to obtain the corresponding VF regions.

The VF sensitivity obtained for each subject was used for the functional measurement analysis. Although 94 eyes qualified for initial inclusion, 2 eyes were subsequently excluded due to the fact that one of the eyes had a very small pupil even with dilation, while the other exhibited unstable fixation. Therefore, the final analysis examined a total of 92 eyes. When trying to determine the association between the GCA thickness and the local loss in VF sensitivity, it is imperative that the macular RGC displacement should also be evaluated [17]. We approximated the average location of the RGCs associated with each VF test point based on equations that were derived from the work of Drasdo et al [18]. Fig 1 shows the location of the VF test points, while Fig 2 shows the location before and after adjusting for the RGC displacement (Figs 1 and 2). Before adjusting for the RGC, we applied the Drasdo et al. [18] displacement model, as most of the inner macular layer areas used for the stimulus points were outside the measurement areas used for the GCA. Our analysis calculated the mean sensitivity of 36 test points (yellow circle), as well as the test points in the lower and upper hemifields (18 test points each). The data collected when using both the VF and OCT in the retinal view were used to determine the structure-function relationships in each of the six sectors. Our analyses demonstrated that the fundus perimetry data were specifically related to the stimulated area. Thus, this indicates that functional measurements of MAIA^TM^ or MP-3 are indeed correlated with the OCT structural measurements in the same hemifield.

### Statistical Analysis

Instead of analyzing the total deviation measured by HFA, this study used MAIA^TM^ and MP-3 for our investigations, as these methods are able to determine the sensitivity in different locations. We calculated the global mean sensitivity values by simply averaging the values.

The correlation between the GCA thickness and the corresponding VF mean sensitivity was examined by using Spearman rank order correlations. An independent Student’s *t*-test and the chi-square test for categorical parameters were used to assess differences between the control and glaucoma groups. Test duration was assessed by Tukey’s HSD test. Intraindividual variation assessments were performed using an intraclass correlation coefficient (ICC). All statistical values are presented as the mean ± standard deviation (SD), with *P* values < 0.05 considered to be statistically significant. All statistical analyses were carried out using SPSS version 19.0 (IBM, New York, NY). Comparisons of the strength of the structure-function association were evaluated by tests of equality of dependent correlation coefficients.

## Results

Table 1 presents the demographic characteristics of the 73 glaucoma patients and 19 healthy subjects enrolled in the study. The grade of the disease, which was based on the standard VF severity grading scale [19], ranged from early to moderate in the glaucomatous eyes of the 73 patients, with 17 (23.9%) classified as early, 24 (32.8%) as moderate, and 32 (43.3%) as severe (Table 2). All of the early, moderate, and severe glaucoma eyes on the 30–2 VFs were classified as abnormal on the 10–2 VFs.

**Table 1:** Clinical characteristics of the study population.

	Glaucoma (n = 73)	Normal (n = 19)	*P* value
Age (y)	63.7±12.9	61.2±12.2	0.59
Gender (M/F)	24/47	5/14	0.50
Refraction (D)	-2.10±2.30	-0.79±2.79	0.12
MD of 10–2 (dB)	-11.8±7.5	-1.7±1.5	<0.001

M: male, F: female, D: diopter, MD: mean deviation.

**Table 2:** OCT measurements and visual field sensitivity an accordance with the severity of glaucoma.

	Early (n = 17)	Moderate (n = 24)	Severe (n = 32)
Age (y)	56.5±13.6	70.7±8.7	62.4±12.7
Gender (Male/Female)	6/11	9/15	10/22
Refraction (D)	-3.39±2.42	-1.18±2.06	-2.10±2.13
Mean deviation (HFA: 30–2, dB)	-3.34±1.36	-9.42±1.57	-18.18±4.07
GCA thickness (um)	65.4±19.0	66.4±8.7	61.0±11.6
Mean sensitivity (dB)			
MAIA	22.8±6.6	19.6±5.2	15.6±5.7
MP-3	22.0±8.1	18.8±5.6	14.4±5.9
HFA	26.8±7.7	23.8±5.8	18.3±7.4

M: male, F: female, D: diopter.

The average sensitivity for each parameter evaluated by the MAIA^TM^, MP-3 and HFA instruments is shown in Table 3. All parameters in the glaucomatous eyes were significantly lower than those of the normal eyes. There was a significant difference in the GCA thickness between the glaucoma and healthy subjects groups (Table 4).

**Table 3:** Macular sensitivity in each instrument.

	Mean Sensitivity, dB		
	Glaucoma	Normal	*P* value
Average			
MAIA	19.0±6.2	25.9±4.2	<0.001
MP-3	18.1±6.9	26.3±4.5	<0.001
HFA	22.9±6.9	31.2±1.6	<0.001
MD of 10–2	-11.89±7.53	-1.65±1.49	<0.001
Superior hemifield			
MAIA	21.7±6.3	26.1±4.0	0.005
MP-3	20.7±7.7	26.1±4.9	0.005
HFA	25.8±7.3	31.5±1.6	0.001
TD of 10–2	-14.04±9.33	-1.77±1.23	<0.001
Inferior hemifield			
MAIA	16.2±8.1	25.6±4.5	<0.001
MP-3	15.4±8.5	26.4±4.2	<0.001
HFA	19.9±9.3	31.0±1.6	<0.001
TD of 10–2	-8.69±7.45	-1.74±1.18	<0.001

MD: mean deviation, TD: total deviation.

**Table 4:** GCA thickness for the average, superior hemifield average, and inferior hemifield average.

	GCA Thickness, μm		
	Glaucoma	Normal	*P* value
Average	64.8±10.6	81.1±6.2	<0.001
Superior hemifield	68.0±12.2	82.4±6.6	<0.001
Inferior hemifield	61.7±11.9	79.7±6.9	<0.001

**Fig 3:**
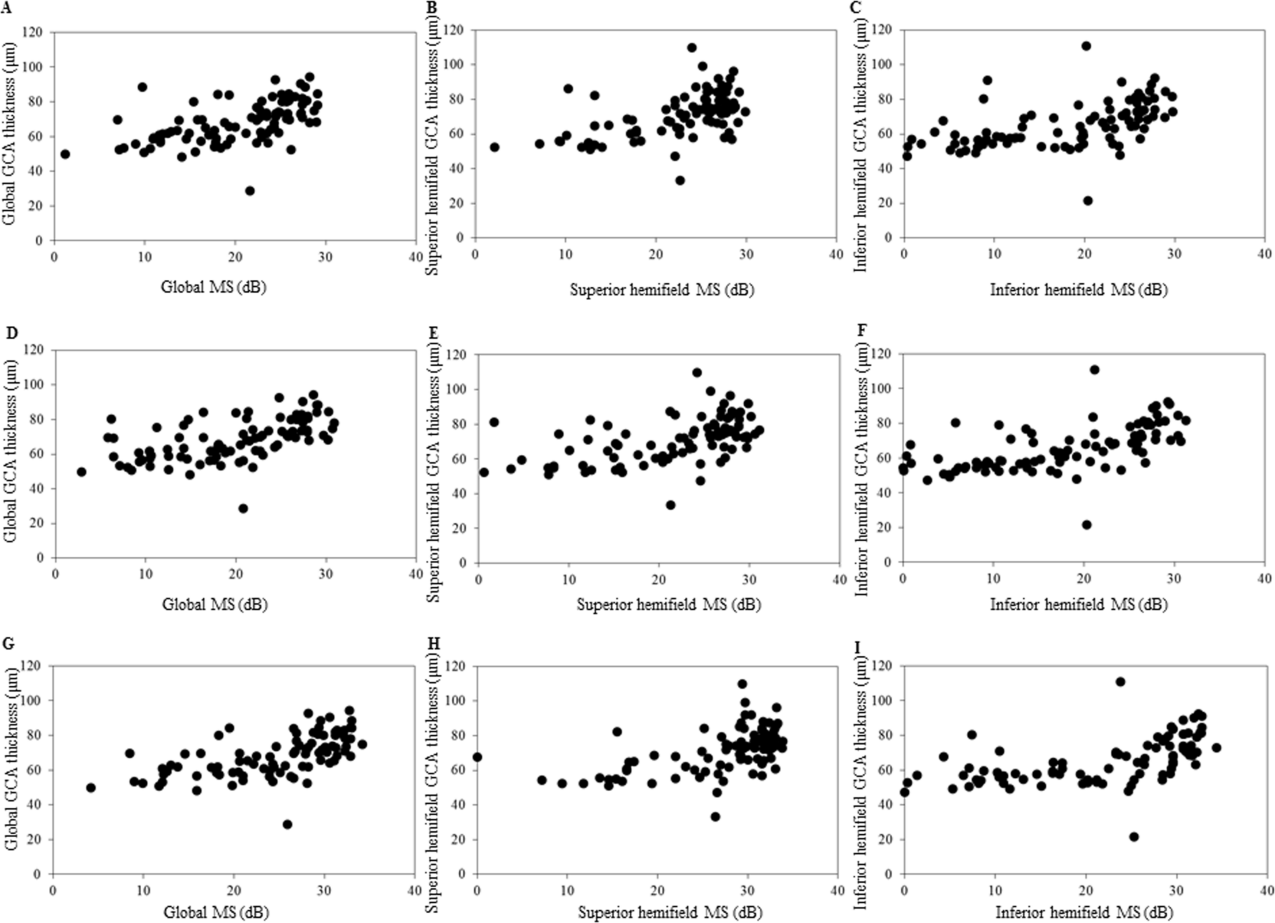
Scatters plots showing the association between the Cirrus HD-OCT thickness parameters and the corresponding retinal sensitivity (decibel) measured either by MAIA^TM^, MP-3, or HFA. (A) Association between the average thickness of the GCA and the macular mean sensitivity as measured by MAIA^TM^. (B) Association between the superior average thickness of the GCA and the superior macular mean sensitivity as measured by MAIA^TM^. (C) Association between the inferior average thickness of the GCA and the inferior macular mean sensitivity as measured by MAIA^TM^. (D) Association between the average thickness of the GCA and the macular mean sensitivity as measured by MP-3. (E) Association between the superior average thickness of the GCA and the superior macular mean sensitivity as measured by MP-3. (F) Association between the inferior average thickness of the GCA and the inferior macular mean sensitivity as measured by MP-3. (G) Association between the average thickness of the GCA and the macular mean sensitivity as measured by HFA. (H) Association between the superior average thickness of the GCA and the superior macular mean sensitivity as measured by HFA. (I) Association between the inferior average thickness of the GCA and the inferior macular mean sensitivity as measured by HFA. Spearman correlation coefficients, *P < 0.001.

The GCA thickness and corresponding VF mean sensitivity were obtained by the MAIA^TM^, MP-3 and HFA and used to evaluate the structure-function relationship (Fig 3 and Table 5). There were no significant differences in the structure-function relationship observed among the three VF measurement instruments. However, the highest correlations for the GCA thickness-VF mean sensitivity for the global and superior hemiretina were found by the HFA, while the highest correlation for the inferior hemiretina GCA thickness-VF mean sensitivity was observed by the MP-3. The highest Spearman correlation coefficient for all of the GCA thickness-VF mean sensitivity measurements in each sector was 0.718 for the HFA measurements in the inferotemporal sector (Table 5).

**Table 5:** Comparison of the strength of structure-function relationship between visual field measurement instruments.

location	Statistic	MAIA	MP-3	HFA
Global	Correlation coefficient	0.627	0.633	0.682
	95% bootstrapped CI	0.515–0.750	0.526–0.732	0.601–0.767
Superior henifield	Correlation coefficient	0.547	0.567	0.602
	95% bootstrapped CI	0.419–0.676	0.454–0.672	0.508–0.721
Inferior hemifield	Correlation coefficient	0.656	0.687	0.642
	95% bootstrapped CI	0.551–0.756	0.583–0.774	0.585–0.757
Superotemporal	Correlation coefficient	0.592	0.603	0.672
	95% bootstrapped CI	0.447–0.691	0.492–0.714	0.570–0.772
Superior	Correlation coefficient	0.426	0.46	0.517
	95% bootstrapped CI	0.275–0.560	0.333–0.577	0.382–0.632
Superonasal	Correlation coefficient	0.417	0.424	0.408
	95% bootstrapped CI	0.265–0.572	0.272–0.554	0.257–0.548
Inferotemporal	Correlation coefficient	0.673	0.691	0.718
	95% bootstrapped CI	0.523–0.767	0.590–0.784	0.623–0.807
Inferior	Correlation coefficient	0.631	0.67	0.651
	95% bootstrapped CI	0.539–0.719	0.569–0.755	0.560–0.730
Inferonasal	Correlation coefficient	0.579	0.616	0.587
	95% bootstrapped CI	0.452–0.716	0.509–0.718	0.485–0.681

CI: confidential interval.

Table 6 shows the intraindividual variations. The ICC values ranged from 0.466 in the superotemporal sector to 0.851 in the inferonasal sector.

**Table 6:** Intraindividual variation of visual field measurements.

Location	ICC (95% CI)
Global	0.752 (0.672–0.819)
Superior henifield	0.684 (0.589–0.766)
Inferior hemifield	0.835 (0.776–0.881)
Superotemporal	0.466 (0.342–0.585)
Superior	0.656 (0.556–0.744)
Superonasal	0.844 (0.789–0.889)
Inferotemporal	0.755 (0.676–0.822)
Inferior	0.842 (0.786–0.887)
Inferonasal	0.851(0.798–0.894)

CI: confidential interval.

Table 7 lists the test durations for the VF measurements in each of the instruments. Among the three instruments, HFA had the shortest test duration for the VF measurements.

**Table 7:** Test duration for each instrument.

	MAIA	MP-3	HFA	*P* value
Test Duratiomn (sec)	532.2±216.6	846.9±155.0	415.5±79.5	<0.001

## Discussion

Macular disorders, such as diabetic macular edema, age-related macular degeneration and macular hole, are commonly evaluated using microperimetry [20–22]. Furthermore, this technique has also been used to assess glaucoma, including peripapillary nerve fiber layer and macular function [10,23–26]. When using microperimetry, MAIA^TM^ or MP-3, these devices are able to automatically record the fixation behavior during the evaluation. In addition, the devices use an autotracking system that makes it possible to adjust stimulus points to predefined retinal positions, thereby ensuring the field testing is reliable. Based on these previous findings and the advantages associated with these devices, we hypothesized that better investigations of the relationship between the function and structure could be achieved when using microperimetry. However, our current study found that evaluations of the macular function by MAIA^TM^ or MP-3 were similar to that for HFA. Since only stable fixation tests were included in the current study, this indicates that there are no additional advantages when using MAIA^TM^ or MP-3 as compared with HFA, which is the standard examination used in glaucoma management.

Previous studies have reported that the strength of the structure-function relationship is related to the individual anatomy and the variations in the subject, the stage of glaucoma in the study sample, the VF scale, OCT segmentation algorithm errors, OCT/perimetry floor effects, test-retest variability, fixation loss, and the regression model used [15,16,27–29]. However, a previous study that examined the test-retest variability found similar results between microperimetry, MP-1 and HFA [30]. We have also examined this relationship and demonstrated that there was a correlation between the GCA thickness and macular visual field sensitivity when measured with MAIA^TM^ [10]. Our previous results indicated that the inferior central VF had a stronger association versus that for the superior central VF. In the current study, our findings not only confirm the results of our previous study, but also show that the results for the MP-3 or HFA are similar to those for MAIA^TM^. Histological studies in humans [31,32] and monkey eyes [33] that examined the central retina have demonstrated that there are more ganglion cells in the superior versus the inferior sectors. Thus, differences in the distribution of the ganglion cells could very well affect the strength of these structure-function relationships.

Mean macular sensitivity evaluated by microperimetry has also been shown to be significantly correlated with the mean HFA results in glaucoma patients [25,26]. These results indirectly support our current study, which demonstrated that the three different VF measurement instruments, MAIA^TM^, MP-3, and HFA, all described similar structure-function relationships in the central VF.

It should be noted, however, that the test duration for the VF measurements in each of the instruments was significantly different. Test durations of the microperimetry devices, MAIA^TM^ or MP-3, were significantly longer than that observed for HFA, due to the fact that the autotracking systems of these devices automatically compensates for eye movement. However, this does not explain why we found the test duration of the MP-3 to be significantly longer than that of MAIA^TM^. One possible explanation may be related to the fact that many of the patients who were examined expressed feeling that the intervals between the stimuli and the stimuli of MP-3 were longer than that of the MAIA^TM^ device.

There were several limitations for our current study. First, we did not find any obvious difference between primary-open angle and normal-tension glaucoma patients with regard to the structure-function relationship. To address this, additional studies that more closely examine the different types of glaucoma will need to be undertaken. Also, since our study did not include any preperimetric glaucoma patients, further studies that examine and compare these patients with regard to the structure-function relationships will need to be undertaken. Second possible limitation is that the controls in our study were undergoing perimetry for perhaps the first time. Experience has been shown to be an important factor when being evaluated by perimetry. Even so, we do not believe that this affected our current results, as our analyses only included reliable tests. Another possible limitation is that learning effects might have influenced the results of our current study. Since the instrument used for the MP-3 examination requires at least a 4 mm pupil diameter, some of the patients had to undergo pupil dilation prior to their evaluation. As a result, it was necessary to set the order of three perimetric tests as HFA, MAIA^TM^, and then MP-3. However, while it has been reported that there is a learning effect associated with HFA [34], there have been no studies to date that have reported finding a learning effect for microperimetry in either subjects with or without retinal diseases [30,35].

## Conclusions

In summary, the measurements of the VF sensitivity in the macular region by MAIA^TM^, MP-3, and HFA were comparable, indicating that these three devices are able to similarly evaluate the structure-function relationship. However, when comparing the structure-function relationship between these instruments, we need take into consideration that intraindividual variations may exist in the subjects being examined by these devices.

## References

[1] Park SC, De Moraes CG, Teng CC, Tello C, Liebmann JM, Ritch R. Initial parafoveal versus peripheral scotomas in glaucoma: risk factors and visual field characteristics. Ophthalmology 2011; 118: 1782–1789. 10.1016/j.ophtha.2011.02.013 21665283

[2] Jung KI, Park HY, Park CK. Characteristics of optic disc morphology in glaucoma patients with parafoveal scotoma compared to peripheral scotoma. Invest Ophthalmol Vis Sci 2012; 53: 4813–4820. 10.1167/iovs.12-9908 22714895

[3] Anctil JL, Anderson DR. Early foveal involvement and generalized depression of the visual field in glaucoma. Arch Ophthalmol 1984; 102: 363–370. 6703983 10.1001/archopht.1984.01040030281019

[4] Fujita K, Yasuda N, Oda K, Yuzawa M. Reading performance in patients with central visual field disturbance due to glaucoma. Nippon Ganka Gakkai Zasshi 2006; 110: 914–918. 17134038

[5] Coeckelbergh TR, Brouwer WH, Cornelissen FW, Van Wolffelaar P, Kooijman AC. The effect of visual field defects on driving performance: a driving simulator study. Arch Ophthalmol 2002; 120: 1509–1516. 12427065 10.1001/archopht.120.11.1509

[6] Ishikawa H, Stein DM, Wollstein G Beaton S, Fujimoto JG, Schuman JS. Macular segmentation with optical coherence tomography. Invest Ophthalmol Vis Sci 2005; 46: 2012–2017. 15914617 10.1167/iovs.04-0335 PMC1939723

[7] Wang M, Hood DC, Cho JS, Ghadiali Q, De Moraes CG, Zhang X, et al Measurement of local retinal ganglion cell layer thickness in patients with glaucoma using frequency-domain optical coherence tomography. Arch Ophthalmol 2009; 127: 875–881. 10.1001/archophthalmol.2009.145 19597108 PMC2987580

[8] Mishra A, Wong A, Bizheva K, Clausi DA. Intra-retinal layer segmentation in optical coherence tomography images. Opt Express 2009; 17:23719–23728. 10.1364/OE.17.023719 20052083

[9] Raza AS, Cho J, de Moraes CG, Wang M, Zhang X, Kardon RH, et al Retinal ganglion cell layer thickness and local visual field sensitivity in glaucoma. Arch Ophthalmol 2011; 129: 1529–1536. 10.1001/archophthalmol.2011.352 22159673 PMC4331118

[10] Sato S, Hirooka K, Baba T, Tenkumo K, Nitta E, Shiraga F. Correlation between the ganglion cell-inner plexiform layer thickness measured with cirrus HD-OCT and macular visual field sensitivity measured with microperimetry. Invest Ophthalmol Vis Sci 2013; 54: 3046–3051. 10.1167/iovs.12-11173 23580483

[11] Mwanza JC, Durbin MK, Budenz DL, Girkin CA, Leung CK, Liebmann JM, et al, Cirrus OCT Normative Database Study Group. Profile and predictors of normal ganglion cell-inner plexiform layer thickness measured with frequency-domain optical coherence tomography. Invest Ophthalmol Vis Sci 2011; 52: 7872–7829. 10.1167/iovs.11-7896 21873658

[12] Mwanza JC, Oakley JD, Budenz DL, Chang RT, Knight OJ, Feuer WJ. Macular ganglion cell-inner plexiform layer: automated detection and thickness reproducibility with spectral domain-optical coherence tomography in glaucoma. Invest Ophthalmol Vis Sci 2011; 52: 8323–8329. 10.1167/iovs.11-7962 21917932 PMC3208140

[13] CenterVue Web site. http://www.Centervue.com/product.php?id=639.

[14] NIDEK CO., LTD. Web site http://www.nidek-intl.com/product/ophthaloptom/diagnostic/dia_retina/mp-3.html.

[15] Anderson RS. The psychophysics of glaucoma: Improving the structure/function relationship. Prog Retin Eye Res 2006; 25: 79–97. 16081311 10.1016/j.preteyeres.2005.06.001

[16] Maddess T. Modeling the relative influence of fixation and sampling errors on retest variability in perimetry. Graefes Arch Clin Exp Ophthalmol 2014; 252: 1611–1619. 10.1007/s00417-014-2751-y 25074042

[17] Hood DC, Raza AS, de Moraes CG, Odel JG, Greenstein VC, Liebmann JM, et al Initial arcuate defects within the central 10 degrees in glaucoma. Invest Ophthalmol Vis Sci 2011; 52: 940–946. 10.1167/iovs.10-5803 20881293 PMC3053114

[18] Drasdo N, Millican CL, Katholi CR, Curcio CA. The length of Henle fibers in the human retina and a model of ganglion receptive field density in the visual field. Vision Res 2007; 47: 2901–2911. 17320143 10.1016/j.visres.2007.01.007 PMC2077907

[19] Hodapp E, Parrish RK II, Anderson DR. Clinical Detection in Glaucoma. St. Louise, MO: Mosby; 1993 pp. 52–61.

[20] Nakamura Y, Mitamura Y, Ogata K, Arai M, Takatsuna Y, Yamamoto S. Function and morphological changes of macula after subthreshold micropulse diode laser photocoagulation for diabetic macular oedema. Eye (Lond) 2010; 24: 784–788. 19680274 10.1038/eye.2009.207

[21] Ozdemir H, Karacorlu M, Senturk F, Karacorlu SA, Uysal O. Microperimetric changes after intravitreal bevacizumab injection for exudative age-related macular degeneration. Acta Ophthalmol 2012; 90: 71–75. 10.1111/j.1755-3768.2009.01838.x 20163371

[22] Ooka E, Mitamura Y, Baba T, Kitahashi M, Oshitari T, Yamamoto S. Foveal microstructure on spectral-domain optical coherence tomographic images and visual function after macular hole surgery. Am J Ophthalmol 2011; 152: 283–290. 10.1016/j.ajo.2011.02.001 21669402

[23] Sato S. Hirooka K, Baba T, Yano I, Shiraga F. Correlation between retinal nerve fiber layer thickness and retinal sensitivity. Acta Ophthalmol 2008; 86: 609–613. 18162063 10.1111/j.1600-0420.2007.01108.x

[24] Orzalesi N, Miglior S, Lonati C, Rosetti L. Microperimetry of localized retinal nerve fiber layer defects. Vis Res 1998; 38: 763–771. 9604104 10.1016/s0042-6989(97)00171-5

[25] Lima VC, Prata TS, DE Moraes CG, Kim J, Seiple W, Rosen RB, et al A comparision between microperimetry and standard achromatic perimetry of the central visual field in eyes with glaucomatous paracentral visual-field defects. Br J Ophthalmol 2010; 94: 64–67. 10.1136/bjo.2009.159772 19692366

[26] Özturk F, Yavas GF, Kusbeci T, Ermis SS. A comparision among Humphrey field analyzer, Microperimetry, and Heidelberg Retina Tomograph in the evaluation of macula in primary open angle glaucoma. J Glaucoma 2008; 17: 118–121. 10.1097/IJG.0b013e31814b97fd 18344757

[27] Leung CK, Chong KK, Chan WM, Yiu CK, Tso MY, Woo J, et al Comparative study of retinal nerve fiber layer measurement by StratusOCT and GDx VCC, II: structure/function regression analysis in glaucoma. Invest Ophthalmol Vis Sci 2005; 46: 3702–3711. 16186352 10.1167/iovs.05-0490

[28] Kim KE, Park KH, Jeoung JW, Kim SH, Kim DM. Severity-dependent association between ganglion cell inner plexiform layer thickness and macular mean sensitivity in open-angle glaucoma. Acta Ophthalmol 2014; 92: e650–656. 10.1111/aos.12438 24836437

[29] Kok PH, van den Berg TJ, van Dijk HW, Stehouwer M, van der Meulen IJ, Mourits MP, et al The relationship between the optical density of cataract and its influence on retinal nerve fiber layer thickness measured with spectral domain optical coherence tomography. Acta Ophthalmol 2013; 91: 418–424. 10.1111/j.1755-3768.2012.02514.x 23106951

[30] Acton JH. Comparing the Nidek MP-1 and Humphery field analyzer in normal subjects. Optom Vis Sci 2011;88: 1288–1297. 10.1097/OPX.0b013e31822b3746 21822159 PMC3204181

[31] Curcio CA, Allen KA. Topography of ganglion cells in human retina. J Comp Neurol 1990; 300: 5–25. 2229487 10.1002/cne.903000103

[32] Curcio CA, Messinger JD, Sloan KR, Mitra A, McGwin G, Spaide RF. Human chorioretinal layer thickness measured in macula-wide, high-resolution histological sections. Invest Ophthalmol Vis Sci 2011; 52: 3943–3954. 10.1167/iovs.10-6377 21421869 PMC3175964

[33] Perry VH, Cowey A. The ganglion cell and cone distributions in the monkey’s retina: implications for central magnification factors. Vision Res 1985; 25: 1795–1810. 3832605 10.1016/0042-6989(85)90004-5

[34] Stewart WC, Hunt HH. Threshold variation in automated perimetry. Surv Ophthalmol 1993; 37: 353–361. 8484168 10.1016/0039-6257(93)90065-f

[35] Weingessel B, Sacu S, Vecsei-Marlovits PV, Weingessel A, Richter-Mueksch S, Schmidt-Erfurth U. Interexaminer and intraexaminer reliability of the microperimeter MP-1. Eye 2009; 23: 1052–1058. 10.1038/eye.2008.237 18670459

